# Preoperative Direct Puncture Embolization of Castleman Disease of the Parotid Gland: A Case Report

**DOI:** 10.3390/curroncol31040151

**Published:** 2024-04-04

**Authors:** Alessandro Pedicelli, Pietro Trombatore, Andrea Bartolo, Arianna Camilli, Esther Diana Rossi, Luca Scarcia, Andrea M. Alexandre

**Affiliations:** 1 UOSA Interventional Neuroradiology, Fondazione Policlinico Universitario Agostino Gemelli IRCCS, 00168 Rome, Italy; alessandro.pedicelli@policlinicogemelli.it (A.P.); andrea.alexandre@policlinicogemelli.it (A.M.A.); 2 U.O.C. Diagnostic Imaging, Interventional Radiology and Neuroradiology, Garibaldi Hospital, 95123 Catania, Italy; pietro.trombatore@gmail.com; 3 Diagnostic and Therapeutic Neuroradiology Unit, IRCCS INM Neuromed, 86077 Isernia, Italy; andreabartolo9@gmail.com; 4 School of Medicine, Catholic University, 00168 Rome, Italy; aricamilli@gmail.com; 5 Division of Anatomic Pathology and Histology, “Agostino Gemelli” School of Medicine, Catholic University of Sacred Heart, 00168 Rome, Italy; esther.rossi@policlinicogemelli.it; 6 Department of Neuroradiology, Henri Mondor Hospital, 94000 Creteil, France

**Keywords:** Castleman, parotid, tumor, embolization, squid

## Abstract

Background: Castleman disease (CD) is an uncommon benign lymphoproliferative disease characterized by hypervascular lymphoid hyperplasia. We present a unique case of unicentric CD of the parotid gland treated by preoperative direct puncture embolization. Case presentation: A 27-year-old female patient was admitted for a right neck mass. Ultrasound examination and MRI scan documented a hypervascular mass within the right parotid gland. Preoperative embolization was performed by direct puncture technique: a needle was inserted into the core of the mass under both ultrasound and fluoroscopic guidance and SQUID 12 was injected into the mass under fluoroscopic control, achieving a total devascularization. Conclusion: Preoperative direct puncture embolization was safe and effective and provides excellent hemostatic control during the surgical operation, limiting the amount of intraoperative bleeding.

## 1. Introduction 

Castleman’s disease (CD) is an uncommon benign lymphoproliferative disease characterized by hypervascular lymphoid hyperplasia, first described by Castleman et al. in 1954 and later in a series of 13 cases in 1956 [1]. 

The incidence of CD has been estimated at 5000 to 6000 patients per year within the US population [2], affecting individuals across all age groups, with the majority falling between 10 and 45 years old, showing no discrepancy by gender [3].

The etiology of CD remains elusive, yet it is hypothesized to stem from an exaggerated immunological response to chronic antigenic stimulation, characterized by an overproduction of interleukin-6.

Based on histologic assessment, three different types have been described: the hyaline-vascular type, the plasma cell type, and the mixed type. The hyaline-vascular type is the most common form, accounting for 80–90% of cases, and usually occurs as a solitary lymphadenopathy [4,5]. 

Two clinical forms of CD are described: the unicentric form and the multicentric form. The unicentric form is characterized by the presence of a single lymphadenopathy, usually asymptomatic, except for the possibility of the compression of adjacent structures due to the size, without abnormal laboratory tests. On the other hand, the multicentric form occurs with poly-lymphadenopathy, multiorgan involvement, and is often associated with systemic symptoms.

The unicentric CD usually occurs as an isolated hypervascular mass. Preoperative endovascular embolization of the main feeders of the lesion is suggested to avoid profuse bleeding during surgery. 

We present a rare case of unicentric CD of the parotid gland in a young woman treated by preoperative direct puncture embolization using SQUID 12. 

## 2. Case

A 27-year-old female patient, with an otherwise unremarkable medical history, was admitted due to the appearance of a slowly expanding mass in the right parotid region.

Clinical examination revealed a conspicuous “mass-like” swelling of the right mandibular region, behind the mandibular angle, with a hard-elastic consistency. The mass was non-tender upon palpation, lacked mobility in both superficial and deep planes, and the overlying skin exhibited a rose-colored appearance. Neurological examination revealed no signs of paralysis of the right cranial nerve VII.

As an initial step, an ultrasound examination was conducted, revealing a solid mass measuring 5 centimeters within the right parotid gland. The mass exhibited intraparenchymal hypervascular Doppler traces, along with low vascular peripheral resistances. An ultrasound-guided fine-needle aspiration was performed, revealing cytological findings of blood fibrin material accompanied by lymphomonocyte elements.

**Figure 1:**
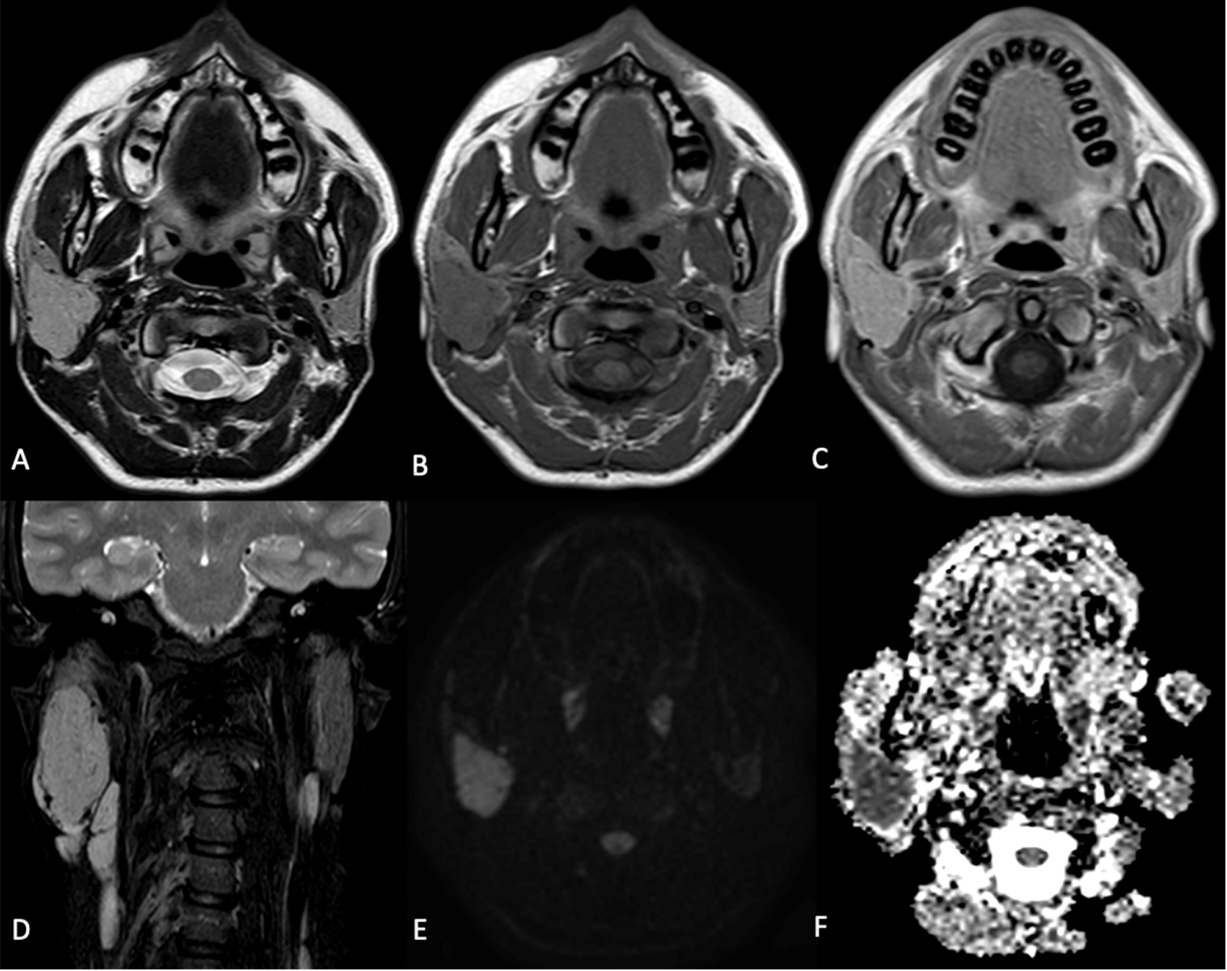
Axial T2WI (**A**), T2WI (**B**) and T1WI after contrast medium (**C**) demonstrate a well-defined solid lesion within the right parotid gland, which is hypo-intense on T1WI, relatively hyperintense in the T2WI image and hyper-intense on T1WI with contrast. There is only a mild diffusion restriction on DWI (**E**) and ADC maps (**F**). On coronal suppressed T2WI (**D**), and on axial T2WI (**A**), we observe some flow-voids images, with expression of high-flow blood vessels, confirmed on T1WI after contrast medium (**C**). Technique: 1.5T MR scanner. 3.0 mm slice thickness.

An MRI scan of the neck obtained approximately three months later revealed an oval hypervascular mass situated in the posterior region of the right parotid gland, positioned posteriorly to the common carotid bifurcation. This mass led to a medial displacement of the internal and external carotid arteries (Figure 1). 

Given the non-univocal cytological characteristics and the atypical presentation including localization, clinical features, and imaging findings, the initial hypothesis leans towards an atypical form of vagal paraganglioma. Following multidisciplinary discussion and considering the evident hypervascular nature of the mass, the patient underwent preoperative endovascular embolization of the lesion prior to surgical excision.

**Figure 2:**
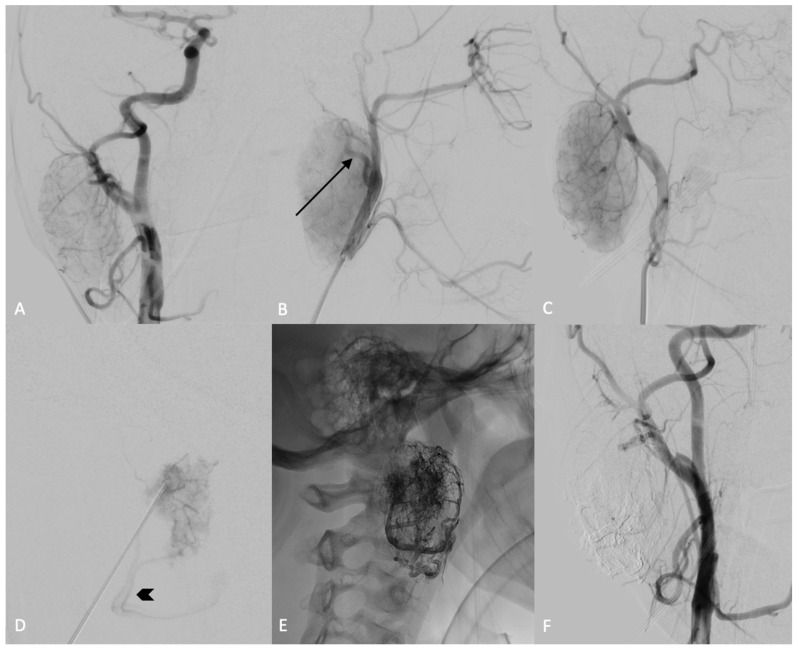
DSA, anterior-posterior view of the right common carotid artery (**A**) and lateral (**B**) and oblique (**C**) views of the right external carotid artery. The injection showed the blush of the lesion laterally to the external carotid artery (**A**–**C**), with evidence of a hypertrophic posterior auricular artery (**B**, black arrow). The lesion was punctured and the needle position was confirmed by angiographic control through injection of the external carotid artery and by an intra-tumoral angiogram obtained after the injection of contrast medium through the needle (**D**); the main venous drainage of the lesion is clearly visible (**D**, black arrowhead). The lesion was slowly filled with SQUID 12, until obtaining complete occlusion (**E**), as confirmed by the absence of blush during the angiographic controls (**F**).

The preoperative embolization was conducted utilizing the direct puncture technique with SQUID 12, employing a biplane flat-panel angiographic system under general anesthesia (Figure 2). A preliminary diagnostic angiographic study was conducted to identify the feeding arteries supplying the mass and to assess for potential extracranial-to-intracranial anastomoses: the digital subtraction angiography (DSA) images revealed a single feeding artery originating from the posterior wall of the right external carotid artery, likely the posterior auricular artery. Additionally, a prominent venous drainage was observed via the facial vein. A 22-gauge needle was inserted into the core of the mass under the guidance of both ultrasound and fluoroscopy. An intra-tumoral angiogram was then conducted through the needle to verify its accurate positioning within the mass. Before injecting the embolic agent, a balloon microcatheter was inflated at the origin of the external carotid artery to prevent retrograde reflux in the internal carotid artery. Subsequently, SQUID 12 was injected through the needle into the mass under fluoroscopic control. At the end of the procedure, the final angiogram showed a total devascularization of the lesion, without any complications. 

**Figure 3:**
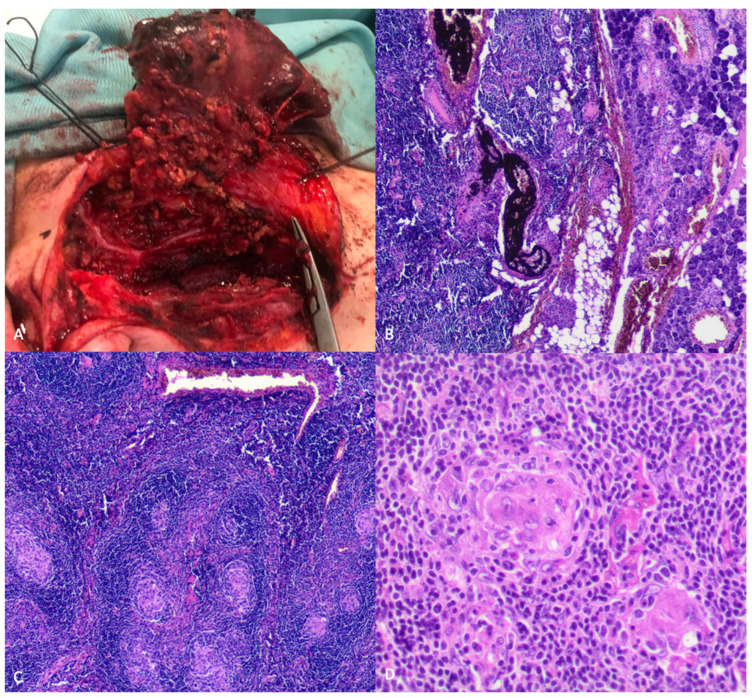
A complete surgical removal of the embolized mass (**A**) was obtained, preserving the facial cranial nerve. The histologic exam showed lymph nodal tissue in the salivary glands, with evidence of previous vascular embolization (**B**) and diffused hyperplastic tissue with germinal centers characterized by hyalinized vessels (**B**,**D**), typically seen in Castleman. A polyclonal proliferation of B lymphocytes is also evident (**B**,**C**).

The surgical removal of the embolized mass was executed two days later. During the surgical intervention, the great auricular nerve was sacrificed, whereas the preservation of the facial cranial nerve was ensured. The healthy parotid gland surrounding the mass was saved. Additionally, three enlarged lymph nodes situated beneath the parotid gland were excised during the procedure. The histological examination conducted on the surgical specimen unveiled a partially embolized hyperplastic intra-parotid lymph node, exhibiting germinal centers with hyalinized vessels, consistent with the hyaline-vascular type of CD (Figure 3). Similar histopathologic findings were identified upon analyzing the remaining lymph nodes as well.

The patient underwent a total body CT scan due to a hypothesis of multicentric CD, which was negative for further localizations of the disease.

Therefore, the final diagnosis was of unicentric CD of the parotid gland. 

## 3. Discussion

Castleman disease (CD) is a benign lymphoproliferative disorder that can impact lymph nodes throughout the body. In a comprehensive series comprising 404 cases of CD, Talat et al. [6] identified the mediastinum as the primary site of involvement, followed by the neck, abdomen, and retroperitoneum.

Cervical localization usually involves retropharyngeal or parapharyngeal neck spaces [7]. The location of the disease within the parotid gland is very rare and very few cases have been reported to date [8]. In a recent literature review by Xiao-dong et al. [9], only 20 cases of parotid gland involvement were reported, with a significant proportion of affected individuals being young adults, consistent with our case.

The diagnosis of CD poses a challenge due to the lack of specific clinical or radiological features. Specifically, the cervical localization of CD presents a considerable challenge in identification due to its potential to mimic other masses in this region, including infectious adenopathy, thyroid carcinoma, Epstein-Barr virus infection, lymphoma, metastatic nodes, paraganglioma, parathyroid adenoma, and schwannoma [10,11].

The primary consideration for a differential diagnosis is with parotid gland tumors, which account for approximately 3–6% of all head and neck neoplasms, with a global incidence ranging from 0.4 to 13.5 per 100,000 persons. Benign tumors typically manifest in the fourth decade of life, whereas malignant tumors tend to occur more frequently in older individuals, often in the sixth or seventh decade [12].

Overall, 80% of parotid gland tumors are benign and pleomorphic adenoma is the most common neoplasm, representing about 60% of parotid gland tumors, followed by monomorphic adenoma, also known as Warthin’s tumor. Despite its benign nature, pleomorphic adenoma can relapse, with recurrence rates of up to 6.8%, and can potentially have a malignant transformation in 5–9.8% of cases. Monomorphic adenoma are less aggressive lesions, arising from remnant lymphoid ducts with little tendency to recur. The difference in behavior between these two tumors carries significant clinical implications for surgical treatment. In cases of monomorphic adenoma, a more conservative surgical approach may be feasible, whereas in pleomorphic adenoma, a more aggressive surgical intervention is often necessary [13].

About 20% of the parotid gland tumors are malignant. The most prevalent malignancy is mucoepidermoid carcinoma, followed by acinic cell carcinoma and adenoid cystic carcinoma. Additionally, lymphomas can occur in the intra-parotid lymph nodes, with an elevated risk observed in individuals with Sjogren syndrome. Lymphoma may present challenges in diagnosis as it can be mistaken for Warthin tumor on cytological examination [14].

However, most of the malignant tumor could be undistinguishable from benign tumors on presentation because all of them present as slow-growing, painless masses. Imaging is not always able to diagnose with certainty the nature of the lesion, so the only investigation that allows us to characterize the lesion is fine-needle aspiration cytology (FNA); however, FNA is inconclusive in almost 6% of cases because of an insufficient sample size or location of the tumor in the deep parotid lobe [12].

When discussing parotid neoplasms, it is also pertinent to consider tumors arising from nerve sheaths, such as schwannomas and neurofibromas, as well as paragangliomas.

A schwannoma is a benign, asymptomatic, encapsulated, and slow-growing tumor. It is a common head and neck tumor and may localized in the parapharyngeal region, most commonly arising from the vagus nerve. Parotid localization is very rare, with an incidence of 0.2–1%. In these cases, they usually arise from the main trunk of the facial nerve. Most facial nerve schwannomas are intra-temporal, with only approximately 9% occurring in the parotid region. Given their rarity among intra-parotid neoplasms and the frequently inconclusive cytology results, achieving a preoperative diagnosis of facial nerve schwannomas can be highly challenging. Only 31.6% who underwent FNA had the correct diagnosis made prior to treatment. However, it is important to take them into account in the differential diagnosis of the parotid gland masses [15,16]. 

Paragangliomas are rare tumors, with an incidence of 1–2 per 100,000 individuals, and only 3% of them arise within the head and neck region. In the majority of cases, paragangliomas are benign; however, they have the potential to become malignant. Malignancy is typically determined when the tumor metastasizes to regional lymph nodes or distant sites. A diagnosis of malignancy cannot be solely determined by cytology or histological findings. The most common localizations in the head and neck are the carotid body (chemodectoma), temporal-bone/middle-ear (glomus jugulare), and the vagus nerves in the neck. Paraganglioma usually presents as an asymptomatic palpable mass in the anterior triangle of the neck, which can easily be confused for a lymph node or other head and neck tumors. The localization of paragangliomas inside the parotid gland is exceedingly rare, with only a few cases reported in the literature. MR and CT imaging typically exhibit specific features, demonstrating a hyper-enhancing soft-tissue mass that may appear homogeneous or heterogeneous, often displaying multiple areas of signal void interspersed with hyperintense foci, giving rise to a “salt-and-pepper” appearance. Angiography commonly reveals an intense tumor blush along with enlarged feeding arteries, underscoring the highly vascular nature of the tumor [17,18,19]. 

The diagnosis of CD can only be reliably achieved through a histological examination of a complete excisional biopsy of the mass. Fine-needle aspiration is frequently inconclusive and may lead to misdiagnosis, such as mistaking it for a Warthin tumor or lymphoma [9]. Additionally, due to the hypervascular nature of the mass, there is a risk of uncontrollable profuse hemorrhage during biopsy, which cannot be ruled out [10].

Imaging is not exhaustive. CT usually shows a well circumscribed homogeneous soft tissue mass with moderate to intense postcontrast enhancement, based on the histologic type: the hyaline vascular type is enhanced more than the plasma cell type due to its greater vascularity. However, these radiological features are common to several other diseases, such as lymphoma or paraganglioma. Recently, it has been suggested that the presence of a non-enhanced stellar scar within the center of the mass, related to dense fibrous tissue, is distinctive [20].

Regarding MR imaging, Takayama et al. [21] suggested that a homogeneously enhanced well-circumscribed mass with hypointense branching structures could be an important diagnostic clue of CD. 

When a CD is suspected, a CT scan of the neck, chest, abdomen, and pelvis is recommended to assess the number of lymph node stations involved and therefore to distinguish between unicentric and multicentric form to provide the patient with the best treatment. In fact, while the gold standard for the treatment of the unicentric form is surgical excision, the multicentric disease benefits from medical therapy. 

Complete surgical resection is the first-line choice in the case of unicentric disease with no risk of recurrences. Clearly, it is necessary to balance the risks and benefits of the surgery, assessing the location of the enlarged lymph node, the possibility of radical resection, and the symptoms caused by the compression of neighboring structures. In the case of an unresectable mass, or in asymptomatic patients with unlikely future compressive symptoms, a careful watch-and-wait approach can be adopted. Other treatment solutions are represented by partial resection to debulk the disease, medical therapy with rituximab, steroids or anti–IL-6 monoclonal antibody therapy, or radiotherapy [22]. 

However, it is crucial to note that surgical excision is frequently associated with significant blood loss due to the hypervascular nature of the tumor [23]. To avoid this potentially serious complication, van Rhee et al. [22] recommend considering the possibility of presurgical embolic devascularization of large masses with a high risk of bleeding (level 2B evidence) [24].

Unfortunately, the challenge of establishing a definitive diagnosis prior to surgery can hinder the recognition of the necessity for embolization. Therefore, it is recommended that a selective arteriography study be considered whenever CT images reveal a hypervascular isolated mass, raising the suspicion of CD or other hypervascular tumors that could potentially benefit from preoperative embolization.

Few cases have been documented regarding the utilization of endovascular preoperative embolization for treating CD, with the majority focusing on cases with a mediastinal localization of the disease [23,25,26,27,28]. Lorenz et al. [29] even reported a case of embolization for a large unresectable mediastinal CD, serving as the definitive treatment without the need for surgery.

Only two cases of a preoperative embolization of unicentric CD of the neck were found in the literature. Sanchez-Ros-Sanchez et al. [30] reported an interesting case of a preoperative endovascular embolization of unicentric CD of the left posterior cervical space using polyvinyl alcohol particles. Newlon et al. [10] described another case of left supraclavicular unicentric CD that was embolized before surgery due to the profuse blood loss during the earlier biopsy. 

We described the first case of a direct puncture preoperative embolization of CD using SQUID 12. To date, no similar cases are reported in the literature. 

Direct puncture embolization for hypervascular head and neck tumors was first described by Casasco et al. [31] in 1994, reporting a good rate of devascularization with a low rate of complications.

The primary advantage offered by this technique is the ability to achieve a complete filling of the tumor microvascular bed, resulting in a more comprehensive occlusion compared to traditional trans-arterial endovascular techniques. Moreover, the direct puncture technique avoids the limitations of trans-arterial embolization, such as the inaccessibility of the feeding arteries and the presence of vasa nervorum and dangerous extracranial-to-intracranial anastomoses, with the risk of inadvertent reflux into the intracranial circulation [32]. Obviously, this technique does not completely exclude the possibility of non-target embolization; for this reason, it is essential to be deeply aware of the vascular anatomy of the lesion and the potential anastomoses, to perform a careful injection of the embolic agent, and to use proper endovascular technique to avoid reflux into the intracranial circulation, such as placing a balloon catheter in the external carotid artery, as in our case.

Another potential risk of direct puncture embolization could be a profuse bleeding at the level of the puncture site given the hypervascular nature of the lesion. However, this is usually quickly managed thanks to the same embolic agents used for the treatment, which can be used both directly through the puncture needle or through intra-arterial catheters, hardly causing clinical consequences for the patient. 

Direct puncture preoperative embolization is widely described in the literature for the treatment of hypervascular head and neck tumors [33,34], such as paraganglioma and juvenile angiofibroma, using different embolic agents such as fibrin glue, polyvinyl alcohol (PVA), nonabsorbable microspheres, absorbable gelatin sponges, and recently, non-adhesive liquid embolic agents (LEA). 

The introduction of non-adhesive LEAs has definitely improved this technique thanks to their lower precipitation time, which leads to a more controlled injection, a deep intra-tumoral penetration, and a reduced risk of non-target embolization. 

Among the non-adhesive LEAs, PHIL, Onyx, and Squid are the most used ones. PHIL (MicroVention, Tustin, California) differs from Onyx and Squid because it is not an ethylene vinyl alcohol (EVOH) copolymer and its radiopacity is not due to tantalum powder but to an iodine component bonded to the copolymer itself; it is available in three different formulations depending on viscosity [35]. Onyx (Medtronic, Irvine, California) was the first commercially available EVOH copolymer and was the gold standard for a long time in the endovascular embolization of vascular malformations. 

SQUID (Emboflu, Gland, Switzerland) is an LEA similar to Onyx, composed by an EVOH copolymer with suspended micronized tantalum powder for radiopacity and DMSO (dimethyl sulfoxide) solvent. There are four different formulations depending on viscosity (18—standard viscosity and 12—low viscosity) and the percentage of dissolved tantalum powder (standard and LD—low dose). While SQUID 18 has similar characteristics to other EVOH copolymers, SQUID 12 is innovative because of its lower viscosity that allows deep penetration into the tumoral parenchyma [36].

## 4. Conclusions

In our experience, the preoperative direct puncture embolization of the vascular supply for this type of mass is safe and effective and can provide excellent hemostatic control during the surgical operation, limiting the amount of intraoperative bleeding.

## Data Availability

Data supporting reported results are available upon request.
